# Variation in population levels of physical activity in European adults according to cross-European studies: a systematic literature review within DEDIPAC

**DOI:** 10.1186/s12966-016-0398-2

**Published:** 2016-06-28

**Authors:** Anne Loyen, Linde Van Hecke, Maïté Verloigne, Ingrid Hendriksen, Jeroen Lakerveld, Jostein Steene-Johannessen, Anne Vuillemin, Annemarie Koster, Alan Donnelly, Ulf Ekelund, Benedicte Deforche, Ilse De Bourdeaudhuij, Johannes Brug, Hidde P. van der Ploeg, on behalf of the DEDIPAC consortium

**Affiliations:** Department of Epidemiology and Biostatistics, VU University Medical Center, EMGO+ Institute for Health and Care Research, De Boelelaan 1089a, 1081 HV Amsterdam, The Netherlands; Department of Public Health, Faculty of Medicine and Health Sciences, Ghent University, De Pintelaan 185, 9000 Ghent, Belgium; Physical activity, Nutrition and Health Research Unit, Department of Movement and Sport Sciences, Faculty of Physical Education and Physical Therapy, Vrije Universiteit Brussel, Pleinlaan 2, 1050 Brussels, Belgium; Department of Movement and Sport Sciences, Faculty of Medicine and Health Sciences, Ghent University, Watersportlaan 2, 9000 Ghent, Belgium; TNO Expertise Centre Lifestyle, Schipholweg 77-89, 2316 ZL Leiden, The Netherlands; Body@Work, EMGO+ Institute for Health and Care Research, VU University Medical Center, van der Boechorststraat 7, 1081 BT Amsterdam, The Netherlands; Department of Sports Medicine, Norwegian School of Sport Sciences, Ullevål Stadion, PO Box 4014, 0806 Oslo, Norway; Faculty of Sport Sciences, EA 4360 APEMAC, University of Lorraine, 30 rue du Jardin Botanique, CS 30156, 54600 Villers-lès-Nancy cedex Nancy, France; Department of Social Medicine, CAPHRI School for Public Health and Primary Care, Maastricht University, PO BOX 616, 6200MD Maastricht, The Netherlands; Department of Physical Education and Sport Sciences, Centre for Physical Activity and Health Research, University of Limerick, Limerick, Ireland; Department of Public and Occupational Health, VU University Medical Center, EMGO Institute for Health and Care Research, van der Boechorststraat 7, 1081 BT Amsterdam, The Netherlands; Sydney School of Public Health, The Charles Perkins Centre (D17), University of Sydney, 2006 NSW Sydney, Australia

**Keywords:** Adults, Assessment methods, Europe, Physical Activity, Prevalence, Review

## Abstract

**Background:**

Physical inactivity is a well-known public health risk that should be monitored at the population level. Physical activity levels are often surveyed across Europe. This systematic literature review aims to provide an overview of all existing cross-European studies that assess physical activity in European adults, describe the variation in population levels according to these studies, and discuss the impact of the assessment methods.

**Methods:**

Six literature databases (PubMed, EMBASE, CINAHL, PsycINFO, SportDiscus and OpenGrey) were searched, supplemented with backward- and forward tracking and searching authors’ and experts’ literature databases. Articles were included if they reported on observational studies measuring total physical activity and/or physical activity in leisure time in the general population in two or more European countries. Each record was reviewed, extracted and assessed by two independent researchers and disagreements were resolved by a third researcher. The review protocol of this review is registered in the PROSPERO database under registration number CRD42014010334.

**Results:**

Of the 9,756 unique identified articles, twenty-five were included in this review, reporting on sixteen different studies, including 2 to 35 countries and 321 to 274,740 participants. All but two of the studies used questionnaires to assess physical activity, with the majority of studies using the IPAQ-short questionnaire. The remaining studies used accelerometers. The percentage of participants who either were or were not meeting the physical activity recommendations was the most commonly reported outcome variable, with the percentage of participants meeting the recommendations ranging from 7 % to 96 % across studies and countries.

**Conclusions:**

The included studies showed substantial variation in the assessment methods, reported outcome variables and, consequently, the presented physical activity levels. Because of this, absolute population levels of physical activity in European adults are currently unknown. However, when ranking countries, Ireland, Italy, Malta, Portugal, and Spain generally appear to be among the less active countries. Objective data of adults across Europe is currently limited. These findings highlight the need for standardisation of the measurement methods, as well as cross-European monitoring of physical activity levels.

**Electronic supplementary material:**

The online version of this article (doi:10.1186/s12966-016-0398-2) contains supplementary material, which is available to authorized users.

## Background

According to the World Health Organization (WHO)’s physical activity recommendations, adults should engage in at least 150 min of moderate-intensity aerobic physical activity per week, or 75 min of vigorous-intensity aerobic activity, or an equivalent combination [1]. Not meeting these recommendations increases the risk of cardiovascular diseases, type 2 diabetes, breast- and colon cancer, and premature death [2, 3]. In 2009, the WHO identified physical inactivity as the fourth leading risk factor for global mortality, causing approximately 6 % of global deaths [2]. A more recent study estimated that physical inactivity was responsible for 9 % of worldwide premature deaths [3].

In 2012, it was estimated that 31.1 % of the adult global population did not meet the physical activity recommendations [4]. Monitoring population levels of physical (in) activity provides the opportunity to track changes over time, identify and target populations with low physical activity levels, and evaluate public health policies and strategies. Internationally comparable data are especially interesting, since they allow cross-country comparisons and benchmarking.

In 2013, twelve European Member States established a Knowledge Hub on DEterminants of DIet and Physical ACtivity (DEDIPAC). One of DEDIPAC’s aims is “enabling a better standardised and more continuous pan-European ‘needs analysis’, i.e. to monitor dietary, physical activity and sedentary behaviours and changes in these behaviours across the life course and within populations to identify targets and target populations for (policy) interventions” [5].

Providing an overview of the existing studies that monitor physical activity across European countries was identified as the first step towards standardisation in population surveillance. In addition, the results of these studies could provide an understanding of the current population levels of physical activity in Europe. A 2010 overview of physical activity surveillance by the WHO Regional Office for Europe concluded that even though population levels of physical activity are frequently monitored across Europe, national surveys were not comparable due to differences in measurement methods while cross-national surveillance efforts were heterogeneous [6]. Hence, the current study provides an updated overview with the sole focus on multi-country studies, in order to enable within-study comparisons of population levels of physical activity across countries.

Four systematic literature reviews have been conjointly performed, focused on 1) sedentary time in youth [7], 2) sedentary time in adults [8], 3) physical activity in youth [9], and 4) physical activity in adults (the current review). The aim of the present review is to a) provide an overview of existing cross-European studies on physical activity in adults (≥18 years), b) describe the variation in population levels of physical activity according to these studies, and c) discuss the impact of study and measurement methods on these population levels.

## Methods

As described in the introduction, this systematic literature review is part of a set of four reviews. Because the four systematic reviews originate from the same project, have similar objectives (although for different behaviours and/or age groups) and share their methodology, the introduction-, methods- and discussion sections of the review articles have obvious similarities. The search, article selection, data extraction and quality assessment were conducted conjointly for all four reviews. Subsequently, the included articles were allocated to the appropriate review article(s). One article could be included in multiple reviews. If an article included both youth (<18 years) and adults (≥18 years) and presented stratified results, those stratified results were used in the appropriate review. If the article did not present stratified results, the article was allocated to the most appropriate review, based on the mean age (and age distribution) of the study sample. Before the search commenced, review protocols were written based on the “Centre for Reviews and Dissemination’s guidance for undertaking reviews in health care” [10], and registered in the PROSPERO database [11]. The review protocol of this review on physical activity in adults is published under registration number CRD42014010334. The reporting of this systematic review adheres to the preferred reporting items of the PRISMA checklist (see Additional file 1).

### Search strategy

The search was conducted in June 2014 and updated on February 29th, 2016. Six databases (PubMed, EMBASE, CINAHL, PsycINFO, SportDiscus and OpenGrey) were searched using similar search strategies, adapted to each database. The following search terms were used: ‘Physical activity’ OR ‘Sedentary behaviour’ AND ‘Europe’ (including all individual country names) AND ‘Countries’/‘Multi-country’/‘International’. Both the index terms and the title and abstract were searched and synonyms (e.g. for physical activity: physically active, physical exercise, etc.) were used. The complete search string can be found in Additional file 2. Based on the in- and exclusion criteria described below, search filters of the databases were used when possible, for example to select the appropriate publication period or language.

In addition, complementary search strategies were used. After the full-text review phase, the reference lists of the included articles were scanned (backward tracking) and a citation search was performed for the included articles (forward tracking) to identify potentially appropriate articles. Also, several experts in the field of physical activity and sedentary behaviour were contacted to provide additional articles. Finally, all authors involved in the four reviews were asked to search their own literature databases for appropriate articles. All additionally retrieved articles underwent the same selection process as the original articles - as described below.

### Article selection

All retrieved records were imported into Reference Manager 12 (Thomson Reuters, New York). Duplicates were hand-searched and removed. Records were included if they were journal articles, reports or doctoral dissertations (further referred to as ‘articles’) written in English. To be included, articles needed to report on observational studies conducted after 01-01-2000 (to avoid reporting outdated data) in the general, healthy population. In addition, articles were only included if they provided data for two or more European countries (as defined by the Council of Europe) [12].

With regard to physical activity, articles were included if they reported total physical activity (e.g. minutes/day or meeting recommendations), and/or physical activity in leisure time. Articles that only reported on transport, occupational or household physical activity were excluded. Both subjective (e.g. questionnaires) and objective (e.g. accelerometers) measures were included.

Three researchers (AL, LVH, MV) were involved in the article selection, data extraction and quality assessment. For the title selection, the three researchers each independently reviewed 1/3 of the titles of the retrieved articles. For the abstract and the full-text selection, data extraction and quality assessment, the three researchers each covered 2/3 of the articles, so that each article was independently reviewed, extracted and assessed by two different researchers. Disagreement between the two researchers was resolved by the third researcher.

### Data extraction

A standardized data extraction file was used to extract data regarding the study characteristics, the study sample, the assessment methods, the reported outcomes, and the findings. We did not request the original data. The complete data extraction file can be found in Additional file 3.

### Quality assessment

A quality score was used to provide a general overview of the quality of the included articles. The ‘Standard quality assessment criteria for evaluating primary research papers from a variety of fields’ [13] was used for the assessment. The checklist consists of fourteen items to be scored ‘Yes’ (2 points), ‘Partial’ (1 point), ‘No’ (0 points) and ‘Not applicable’. The summary score was calculated as follows: Total sum ((number of ‘Yes’ x 2) + (number of ‘Partial’ x 1)) / Total possible sum (28 – (number of ‘Not applicable’ x 2)). This instrument was chosen because it provides the opportunity to assess and compare the quality of different study designs, focuses on both the research and the reporting, and allows researchers to indicate that an item is not applicable, without affecting the total quality score. The complete quality assessment file can be found in Additional file 4.

## Results

**Fig. 1:**
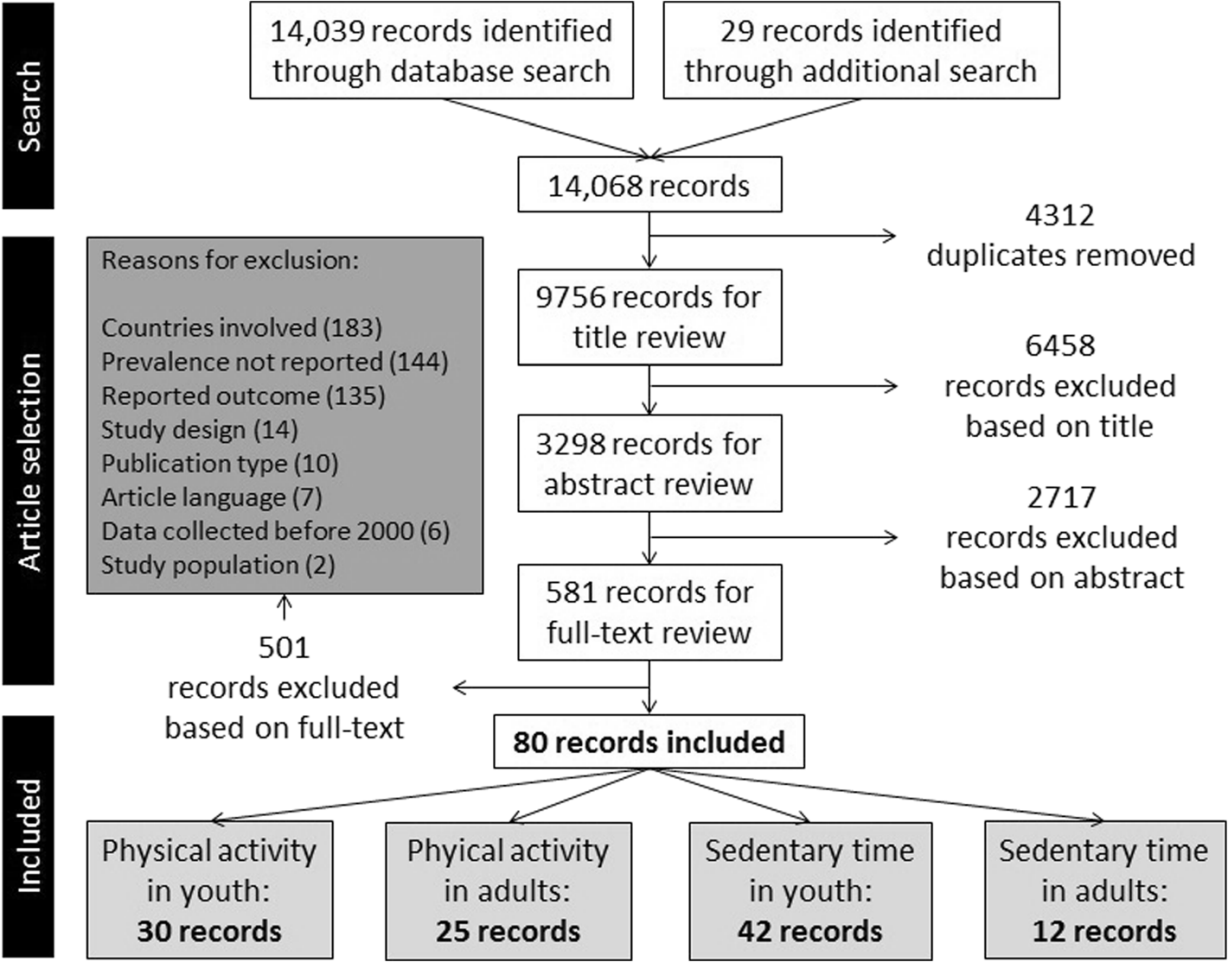
Flowchart of the combined review process

The flowchart of the combined review process for all four reviews is shown in Fig. 1. The search (original and update combined) resulted in 14,068 records (14,039 through the database search and 29 through the additional search), of which 9756 were unique. 6458 records were excluded based on their title, and an additional 2717 based on their abstract, leaving 581 records for the full-text review phase. In this phase, 501 records were excluded, mainly because the studies did not include at least two European countries (N = 183); because the prevalence numbers were not reported per country (N = 144); or because the reported outcomes were not relevant (N = 135). The remaining 80 records were eligible for inclusion. In the current review on physical activity in adults, 25 records were included [4, 14–37].

### Overview of the existing cross-European studies on physical activity in adults

The study and sample characteristics of the included articles are shown in Table 1. All articles were published between 2002 and 2016. Multiple articles reported on the Eurobarometer surveys [14–18], the International Prevalence Study (IPS) [19–21], the WHO global health observatory [4, 22, 23], the European Prospective Investigation into Cancer and nutrition (EPIC) [24, 25], the International Physical activity and the Environment Network (IPEN) [26, 27], and the World Health Survey (WHS) [28, 29]. Except for one longitudinal study [37], all studies used a cross-sectional design. The quality score ranged from 0.59 to 1.00 (on a scale from 0 to 1). The number of countries involved in each study ranged from 2 to 35 and the number of participants from 321 to 274,740. Most studies included a broad age group, but one study only included older adults between 50 and 64 years old [24], while another study focused on young adults aged 21 and 25 [37]. In addition, one study only included women [25], while another study sample only included parents and comprised 82 % women [34]. All studies used questionnaires to assess physical activity, except for two studies (reported in three articles) using ActiGraph accelerometers [26, 27, 37]. The percentage of people meeting, and not meeting, the physical activity recommendations were the most frequently reported outcome variables. None of the included articles reported data from Armenia, Azerbaijan, Former Yugoslav Republic of Macedonia, Republic of Moldova, Montenegro, and the microstates Andorra, Liechtenstein, Monaco and San Marino.

**Table 1:** Study information and sample characteristics of the articles included in the systematic review

Article	Study	Study design	Quality score (0–1)	Number of European countries	Number of European participants	Demographics				Physical activity assessment method	Reported physical activity outcome variables
						Age (range)	Gender (% female)	Level of education	BMI (mean)		
Eurobarometer (EB)											
Friedenreich et al. (2010) [14]	EB 58.2 (2002)	CS	0.59	15	n.r.	15+	n.r.	n.r.	n.r.	Questionnaire; IPAQ-short	% insufficiently active
Rutten & Abu-Omar (2004) [15]	EB 58.2 (2002)	CS	0.85	15	16,230	15+	54 %	n.r.	n.r.	Questionnaire; IPAQ-short	MET-hours/week
Sjöström et al. (2006) [16]	EB 58.2 (2002)	CS	0.91	15	n.r.	15+	n.r.	n.r.	n.r.	Questionnaire; IPAQ-short	% sufficiently active
Eurobaro-meter 64.3 (2006) [17]	EB 64.3 (2005)	CS	0.75	29	29,195	15+	n.r.	n.r.	n.r.	Questionnaire; IPAQ-short	Days/week and minutes/day MPA and VPA
Gerovasili et al. (2015) [18]	EB 80.2 (2013)	CS	0.86	28	19,978	18-69	n.r.	n.r.	n.r.	Questionnaire; IPAQ-short	% (in) sufficiently active and MET-minutes/week
International Prevalence Study (IPS)											
Bauman et al.(2009) [19]	IPS	CS	0.95	7	17,749	18-65	48-59%^a^	3-58 % >13 years edu^a^	n.r.	Questionnaire; IPAQ-short	% low; moderate; high physical activity levels
Ding et al. (2013) [20]	IPS	CS	0.95	4	2,533	18-65	44-53%^a^	n.r.	n.r.	Questionnaire; IPAQ-short	% meeting physical activity recommendations
Sallis et al. (2009) [21]	IPS	CS	1.00	4	2,574	18-65	56 %	45 % >13 years edu	n.r.	Questionnaire; IPAQ-short	% meeting guidelines for physical activity
World Health Organization global health observatory											
Hallal et al. (2012) [4]	/	CS	0.75	35	270,862	15+	n.r.	n.r.	n.r.	Questionnaire; unspecified	% physically inactive
Kahan (2015) [22]	/	CS	0.77	2	9,969	15+	52 %	n.r.	n.r.	Questionnaire; IPAQ, GPAQ, or similar	% physical inactivity
Papandreou & Tuomilehto (2013) [23]	Seven Countries Study	CS	0.73	5	n.r.	15+	n.r.	n.r.	26	Questionnaires; unspecified	% physically inactive
European Prospective Investigation into Cancer and Nutrition (EPIC)											
Haftenberger et al. (2002) [24]	EPIC	CS	0.90	9	236,386	50-64	67 %	n.r.	n.r.	Questionnaire; Standardized lifestyle questionnaire	Hours/week of total recreational activity
Lahmann et al. (2009) [25]	EPIC	CS	0.95	9	274,740	n.r.	100 %	n.r.	n.r.	Questionnaire; Standardized lifestyle questionnaire	MET-hours/week of combined household and recreational physical activity
International Physical activity and the Environment Network (IPEN)											
Cerin et al. (2014) [26]	IPEN	CS	0.91	5	2,166	18-66	54 %	50 % college or higher	n.r.	Accelerometer; ActiGraph	Minutes/day MVPA and % meeting guidelines
Van Dyck et al. (2015) [27]	IPEN	CS	0.91	5	2,166	18-66	53 %	52 % college or higher	26	Accelerometer; ActiGraph (several models)	Counts/minute and minutes/day MVPA
World Health Survey (WHS)											
Atkinson et al. (2016) [28]	World Health Survey	CS	0.91	9	23,527	18-69	n.r	n.r	n.r	Questionnaire; IPAQ-short	% physically inactive
Guthold et al. (2008) [29]	World Health Survey	CS	0.90	12	24,995	18-69	56 %	n.r.	n.r.	Questionnaire; IPAQ-short	% physically inactive
Other studies											
Alkerwi et al. (2015) [30]	/	CS	0.91	3	3,133	18-69	51 %	35 % tertiary edu	n.r.	Questionnaire; IPAQ-long	% low; moderate, high physical activity levels and % compliance
Bamana et al.(2008) [31]	EUPASS	CS	1.00	7	4,231	18+	57 %	65 % working	24	Questionnaire; IPAQ-short	% low; moderate; high physical activity levels
Bourdeau-dhuij et al. (2005) [32]	/	CS	0.95	2	526	18+	65-66%^a^	40-44 % higher edu^a^	23-26^a^	Questionnaire; IPAQ-long	Minutes/week of all MVPA in leisure time and total MVPA
Hughes et al. (2015) [33]	/	CS	0.70	2	n.r	25-84	n.r.	n.r.	n.r.	Questionnaire; IPAQ	% physical inactivity
Jimenez-Pavon et al. (2012) [34]	ENERGY	CS	0.82	7	5,296	n.r.	82 %	n.r.	n.r.	Questionnaire; ENERGY parent questionnaire	% meeting physical activity guidelines
Lakerveld et al. (2015) [35]	SPOT-LIGHT	CS	0.95	5	6,037	n.r.	56 %	54 % high edu level	25	Questionnaire; IPAQ-long (adapted)	Minutes/day of MVPA in leisure time
Marques et al. (2015) [36]	ESS	CS	0.82	27	n.r.	n.r.	54 %	22.8 % superior edu	n.r.	Questionnaire; single item	% attained physical activity
Ortega et al. (2013) [37]	EYHS	LT	0.91	2	321	21 and 25	59-63%^a^	n.r.	20-21^a^	Accelerometer; ActiGraph	Minutes/day MVPA

*BMI* Body Mass Index, *EUPASS* European Physical Activity Surveillance System, *ENERGY* EuropeaN Energy balance Research to prevent excessive weight Gain among Youth, *SPOTLIGHT* Sustainable Prevention of Obesity Through Integrated Strategies, *ESS* European Social Survey, *EYHS* European Youth Heart Study, *CS* Cross-sectional, *LT* Longitudinal, *n.r.* not reported, *yrs* years, *edu* education, *IPAQ* International Physical Activity Questionnaire, *GPAQ* Global Physical Activity Questionnaire, *MET* metabolic equivalent, *MPA* moderate physical activity, *VPA* vigorous physical activity, *MVPA* moderate-to-vigorous physical activity

^a^These publications only presented stratified demographics. The numbers shown here represent the range

### Variation in population levels of physical activity in European adults

As discussed, several articles reported on data from the same study. To avoid presenting results from the same data twice, we used one article per study to describe reported physical activity levels. This selection was based on the information in the article and the similarities with the other articles. Thus, eight articles presenting duplicate results were excluded [14, 15, 20, 21, 23, 25, 27, 28]. The following section will discuss the seventeen remaining articles.

Table 2 provides an overview of the levels of physical activity in adults across European countries, as a summary of the results reported in the included articles. To enable comparison across studies, we harmonised these results where this was possible. For example, some articles [4, 22, 29, 33] reported the percentage of participants *not* meeting the physical activity recommendations. After checking for missing values, we reversed those numbers. Another article [17] reported days/week and minutes/day of moderate physical activity (MPA) and vigorous physical activity (VPA), which we converted into minutes/week moderate-to-vigorous physical activity (MVPA) using the following formula: ((days/week VPA * min/day VPA) + (days/week MPA * min/day MPA)). Finally, two articles [26, 37] reported MVPA minutes per day instead of per week, which we multiplied by seven to calculate minutes per week.

**Table 2:** Levels of physical activity in adults across European countries. This table displays a summary of the results reported in the articles included in the systematic review

	Total physical activity				Physical activity in leisure time	
	% meeting recommendations [4, 16, 18, 22, 26, 29, 30, 33, 34, 36]^a^	Mean min/week MVPA [17, 26, 32, 37]^b,c^	% low; moderate; high physical activity [19, 30, 31]	Mean MET-min/week [18]	Mean min/week MVPA in leisure time [32, 35]	Mean hours/week recreational activity [24]^d^
Albania	61 % [22] 76 %(M); 83 %(F) [36]					
Austria	65 % [4] 26 % [16] 76 % [18]	499 [17]		2428		
Belgium	57 % [4] 25 % [16] 68 % [18] 16 % [26] 71 % [30] 16 % [34] 68 %(M); 68 %(F) [36]	408 [17] 252 [26] 601 [32]	43 %; 27 %; 30 % [19] 28 %; 36 %; 36 % [30]	1981	152 [32] 264 [35]	
Bosnia and Herzegovina	66 % [4] 88 %(M); 83 %(F) [29]					
Bulgaria	73 % [4] 67 % [18] 78 %(M); 75 %(F) [36]	675 [17]		2054		
Croatia	76 % [4] 78 % [18] 90 %(M); 92 %(F) [29]	775 [17]		2546		
Cyprus	45 % [4] 46 % [18] 78 %(M); 77 %(F) [36]	378 [17] (RoC) 196 [17] (TCC)		1321		
Czech Republic	75 % [4] 73 % [18] 30 % [26] 89 %(M); 93 %(F) [29] 63 %(M); 68 %(F) [36]	607 [17] 322 [26]	10 %; 27 %; 63 % [19]	2348		
Denmark	65 % [4] 34 % [16] 82 % [18] 24 % [26] 56 %(M); 52 %(F) [36]	468 [17] 280 [26]		2198		8.8-10.5 *(50–64 years old)*
Estonia	83 % [4] 80 % [18] 96 %(M); 95 %(F) [29] 70 %(M); 66 %(F) [36]	824 [17] 301 (M); 245 (F) [37] *(25 years old)*		2910		
Finland	62 % [4] 33 % [16] 84 % [18] 53 %(M); 53 %(F) [36]	394 [17]	12 %; 29 %; 58 % [31]	2200		
France	68 % [4] 24 % [16] 71 % [18] 74 % [30] 70 %(M); 75 %(F) [36]	259 [17]	26 %; 40 %; 34 % [30] 19 %; 29 %; 52 % [31]	2270	230 [35]	11.4-12.2 *(50–64 years old)*
Georgia	78 % [4] 7 %(M); 9 %(F) [29]					
Germany	72 % [4] 40 % [16] 84 % [18] 60 %(M); 68 %(F) [36]	637 [17]	10 %; 24 %; 66 % [31]	2751		12.8-16.1 *(50–64 years old)*
Greece	84 % [4] 37 % [16] 62 % [18] 16 % [34]	667 [17]		1611		10.9-11.2 *(50–64 years old)*
Hungary	74 % [4] 67 % [18] 92 %(M); 92 %(F) [29] 26 % [34] 64 %(M); 70 %(F) [36]	593 [17]		2229	287 [35]	
Iceland	46 %(M); 41 %(F) [36]					
Ireland	47 % [4] 29 % [16] 75 % [18] 59 % [33] 66 %(M); 62 %(F) [36]	191 [17]		1926		
Italy	45 % [4] 26 % [16] 53 % [18] 48 %(M); 56 %(F) [36]	212 [17]	30 %; 46 %; 25 % [31]	1259		1.0-10.3 *(50–64 years old)*
Latvia	68 % [4] 81 % [18]	772 [17]		3027		
Lithuania	77 % [4] 76 % [18] 55 %(M); 51 %(F) [36]	635 [17]	15 %; 33 %; 52 % [19]	2379		
Luxembourg	52 % [4] 36 % [16] 82 % [18] 82 % [30]	376 [17]	18 %; 27 %; 54 % [30]	2174		
Malta	28 % [4] 51 % [18]	45 [17]		1379		
Netherlands	82 % [4] 44 % [16] 85 % [18] 28 % [34] 67 %(M); 67 %(F) [36]	960 [17]	16 %; 28 %; 57 % [31]	2634	368 [35]	14.9-20.2 *(50–64 years old)*
Norway	56 % [4] 57 % [34] 53 %(M); 53 %(F) [36]		26 %; 34 %; 40 % [19]			
Poland	72 % [4] 56 % [18] 72 %(M); 75 %(F) [36]	599 [17]		1461		
Portugal	49 % [4] 33 % [16] 49 % [18] 68 %(M); 63 %(F) [36]	213 [17] 406 [32]	26 %; 29 %; 45 % [19]	1139	147 [32]	
Romania	61 % [4] 72 % [18]	599 [17]		2373		
Russian Federation	79 % [4] 93 %(M); 95 %(F) [29] 59 %(M); 57 %(F) [36]					
Serbia	32 % [4]					
Slovak Republic	78 % [4] 72 % [18] 84 %(M); 93 %(F) [29] 80 %(M); 85 %(F) [36]	808 [17]		2156		
Slovenia	70 % [4] 72 % [18] 90 %(M); 86 %(F) [29] 47 % [34] 73 %(M); 72 %(F) [36]	688 [17]		2019		
Spain	50 % [4] 25 % [16] 80 % [18] 31 % [26] 73 %(M); 67 %(F) [29] 29 % [34] 46 %(M); 43 %(F) [36]	155 [17] 357 [26]	24 %; 36 %; 40 % [19] 29 %; 33 %; 38 % [31]	2166		7.3-17.3 *(50–64 years old)*
Sweden	56 % [4] 23 % [16] 88 % [18] 52 %(M); 58 %(F) [36]	187 [17] 322 (M); 280 (F) [37] *(21 year old)*	24 %; 37 %; 39 % [19]	2415		5.8-5.9 *(50–64 years old)*
Switzerland	55 %(M); 62 %(F) [36]					
Turkey	44 % [4] 44 % [22] 72 %(M); 58 %(F) [29]	253 [17]				
Ukraine	82 % [4] 95 %(M); 96 %(F) [29] 70 %(M); 74 %(F) [36]					
United Kingdom	37 % [4] 29 % (GB) [16] 76 % [18] 19 % [26] 58 % (NI) [33] 37 % (SC) [33] 65 %(M); 64 %(F) [36]	242 [17] 259 [26]	27 %; 41 %; 32 % (EN) [31]	2543	265 [35]	12.9-15.4 *(50–64 years old)*

*Min* minutes, *MVPA* Moderate-to-Vigorous Physical Activity, *MET* metabolic equivalent, *M* Males, *F* Females, *RoC* Republic of Cyprus, *TCC* Turkish Cypriot Community, *yrs* years, *GB* Great Britain, *NI* Northern Ireland, *SC* Scotland, *EN* England. ^a^. Some studies [4, 22, 29, 33] reported the percentage of participants NOT meeting the physical activity recommendations. We reversed those numbers to enable comparison. ^b^. Study [17] reported days/week and minutes/day of moderate physical activity (MPA) and vigorous physical activity (VPA) separately. We used the following formula to arrive at min/week MVPA: ((days/week VPA * min/day VPA) + (days/week MPA * min/day MPA)). ^c^. Studies [26, 37] reported min per day MVPA instead of per week. To enable comparison, we multiplied these numbers by 7 to calculate minutes per week. ^d^. This study reported the results stratified by study center and gender. The numbers shown here are the lowest and highest results per country

Ten articles reported the percentage of participants meeting the physical activity recommendations. The lowest percentages (7 % in males and 9 % in females) were found in Georgia, while the highest percentage (96 %) was found in Ukrainian females and Estonian males [29]. Four articles reported the time spent in MVPA, which ranged from 45 min/week in Malta to 960 min/week in the Netherlands [17]. Three articles reported the percentage of participants with low, moderate and high physical activity levels, and the percentage of participants with high physical activity levels varied from 25 % in Italy to 66 % in Germany [31]. MET-minutes/week ranged from 1139 in Portugal to 3027 in Latvia [18]. The Portuguese reported 147 min/week of MVPA in leisure time [32], whereas the Dutch reported 368 min/week [35]. Finally, Italian elderly reported the least hours of recreational physical activity per week(1.0), whereas Dutch elderly reported the most (20.2) [24].

**Fig. 2:**
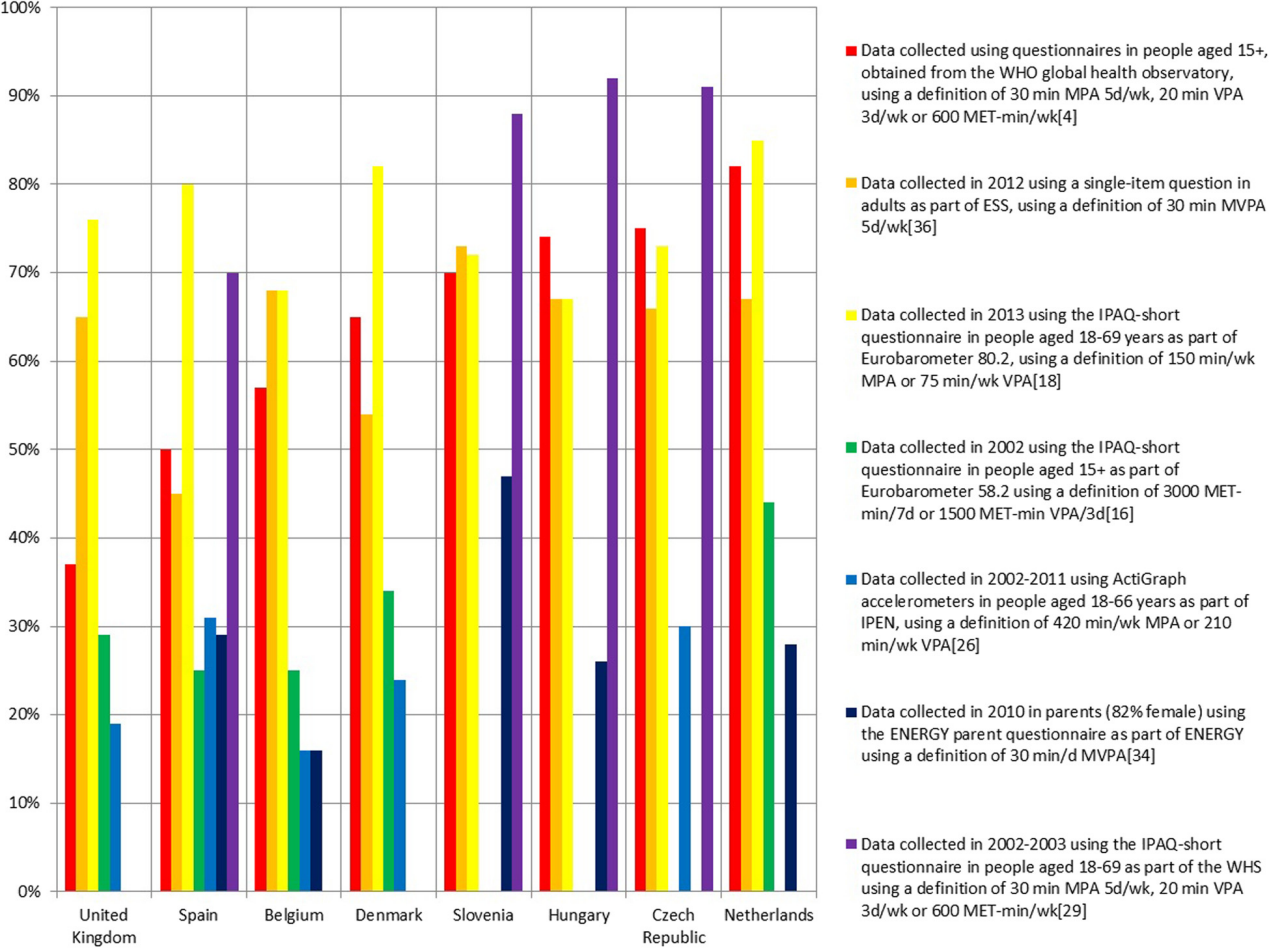
The percentage of adults meeting physical activity recommendations across countries based on different articles. WHO = World Health Organization; Min = minutes; MPA = Moderate intensity physical activity; d = days; wk = week; VPA = Vigorous intensity physical activity; MET = Metabolic Equivalent; MVPA = Moderate-to-vigorous physical activity; ESS = European Social Survey; IPAQ = International Physical Activity Questionnaire; IPEN = International Physical activity and the Environment Network; ENERGY = European Energy balance Research to prevent excessive weight Gain among Youth; WHS = World Health Survey. The World Health Survey and European Social Survey stratified their results by gender. We calculated the (weighted) average

In order to provide a more accessible overview of the results, Fig. 2 shows the percentage of participants meeting the physical activity recommendations in eight different countries, based on seven different studies. This outcome was chosen because it was reported most often; these countries and studies were included because they provided most data points. Because the WHS [29] and ESS [36] results were stratified by gender, we calculated the (weighted) mean. In general, the WHS reports the highest percentages [29], while IPEN [26] and ENERGY [34] report the lowest percentages of participants meeting the physical activity recommendations. The three studies using the IPAQ-short questionnaire [16, 18, 29] show quite some differences across countries. Overall, the greatest variety can be found in Hungary, where the reported percentage of participants meeting the physical activity recommendations ranges from 26 % up to 92 %.

**Fig. 3:**
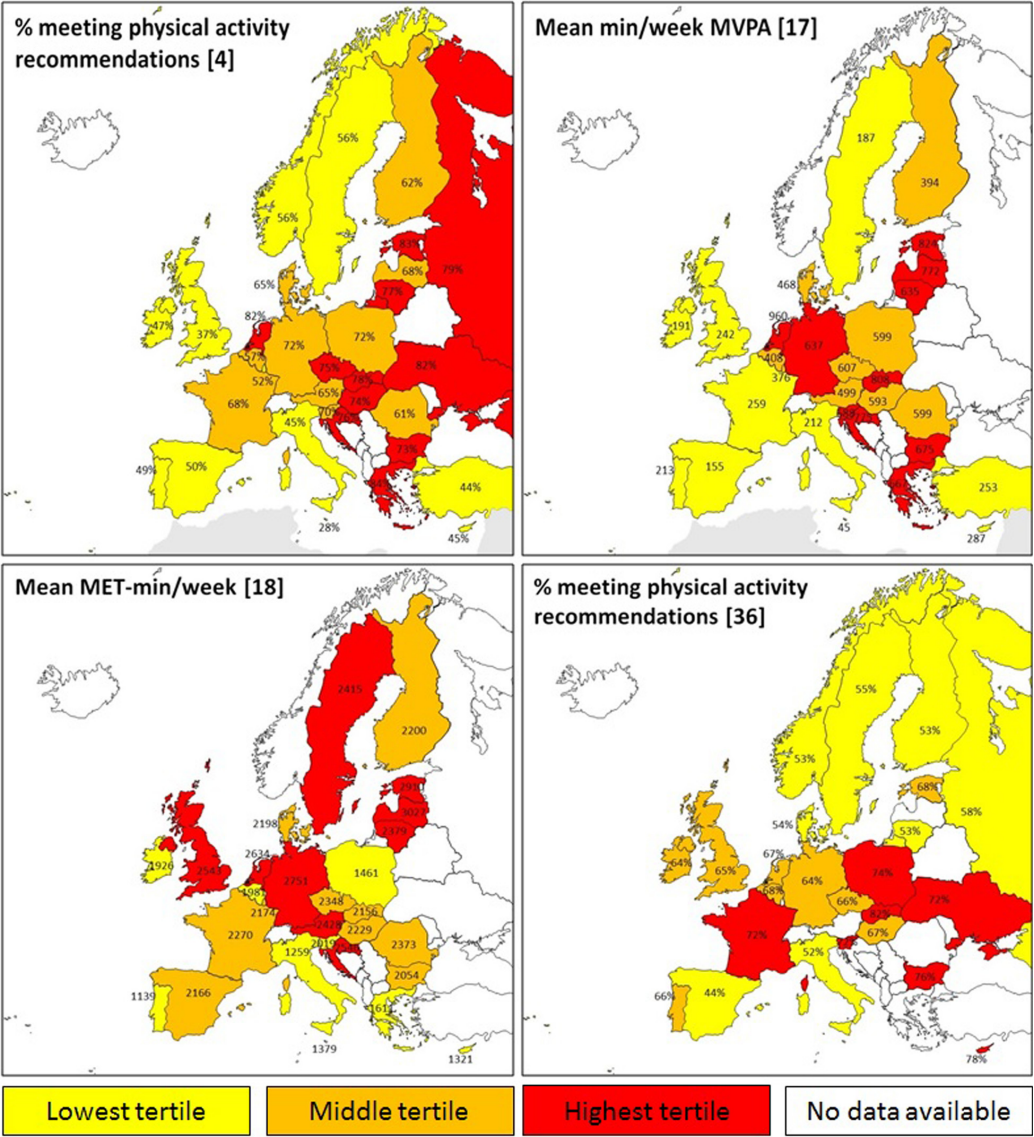
The distribution of physical activity levels across Europe, showing **a**) the percentage of participants meeting the physical activity recommendations [4], **b**) the mean minutes/week of MVPA [17], **c**) the mean MET-minutes/week [18], and **d**) again the percentage meeting the recommendations [36]. All variables were self-reported. The yellow, orange and red colouring represent the lowest, middle and highest tertiles, respectively, based on the reported outcome variable. The countries marked white had no available data in (the majority of) the studies. Designed by Showeet.com

In addition, we constructed four maps of Europe based on the largest studies included in this review, which are shown in Fig. 3. They show the distribution of a) the percentage of participants meeting the physical activity recommendations [4], b) the mean minutes/week of MVPA [17], c) the mean MET-minutes/week [18], and d) the percentage meeting the recommendations [36]. All variables were self-reported. The colourings represent the lowest, middle and highest tertiles, respectively, based on the reported outcome variables. In general, the geographical patterns of the tertiles seem to be reasonably coherent between the four studies, with Italy consistently belonging to the lowest tertile in all studies, and Ireland, Malta, Portugal and Spain belonging to the lowest tertile in the majority of the studies.

### Variation in assessment methods and reported outcome variables

An overview of the assessment methods and the reported outcomes of the included articles is shown in Table 3. In this overview, all twenty-five included articles are considered again, in order to give a complete overview. The articles reporting on the same study are indicated in the table. Twenty-one articles focused on total physical activity, three articles focused on physical activity in leisure-time [24, 25, 35], and one study studied both [32]. In total, nine assessment methods were used to report twelve different outcomes. Six studies (reported in eleven articles) used the IPAQ-short questionnaire [14–21, 28, 29, 31]. Fifteen articles (based on ten studies) reported the percentage of participants who either were or were not sufficiently active/meeting the physical activity recommendations [4, 14, 16, 18, 20–23, 26, 28–30, 33, 34, 36]. These fifteen articles used seven different ways of operationalizing physical (in) activity. While some of the articles that reported on the same study reported identical outcome variables, other articles that reported on the same study show differences in their reported outcomes and/or the operationalization of physical (in) activity.

**Table 3:** Assessment methods and reported outcome variables in the articles included in the systematic review

	N	Reference (s)
**Studies with multiple articles**		
EB 58.2	3	EB [14–16]
IPS	3	IPS [19–21]
WHO global health observatory	3	WHO [4, 22, 23]
EPIC	2	EPIC [24, 25]
IPEN	2	IPEN [26, 27]
WHS	2	WHS [28, 29]
**Assessment method**		
*Questionnaire*	22	EB [14–16], IPS [19–21], WHO [4, 22, 23], EPIC [24, 25], WHS [28, 29], [17, 18], [30–36]
IPAQ-short	11	EB [14–16], IPS [19–21], WHS [28, 29], [17, 18], [31]
IPAQ-long	3	[30, [32], [35]
IPAQ-unknown	1	[33]
Standardized lifestyle questionnaire	2	EPIC [24, 25]
ENERGY parent questionnaire	1	[34]
Single-item	1	[36]
Unspecified/multiple	3	WHO [4, 22, 23]
*Accelerometer (Actigraph)*	3	IPEN [26, 27], [37]
≥1952 counts per minute	2	IPEN [26, 27]
≥2000 counts per minute	1	[37]
**Reported outcomes**		
*Total physical activity*	22	EB [14–16], IPS [19–21], WHO [4, 22, 23], IPEN [26, 27], WHS [28, 29], [17, 18], [30–34], [36, 37]
% insufficiently active	8	EB [14], WHO [4, 22, 23], WHS [28, 29], [18, 33]
MET-hours/week	1	EB [15]
% sufficiently active	8	EB [16, 18] IPS [20, 21], IPEN [26, 30, 34, 36]
Days/week and minutes/day MPA and VPA	1	[17]
MET-minutes/week	1	[18]
% low; moderate; high physical activity	3	IPS [19, 30, 31]
Minutes/day MVPA	4	IPEN [26, 27, 32, 37]
Counts/min	1	IPEN [27]
*Physical activity in leisure time*	4	EPIC [24, 25, 32, 35]
Hours/week total recreational activity	1	EPIC [24]
MET-hours/week household and recreational PA	1	EPIC [25]
Minutes/week MVPA in leisure time	1	[32]
Minutes/day MVPA in leisure time	1	[35]
**Operationalization of physical (in)activity**		
3000 MET-min/7 days, or 1500 MET-min VPA/3 days	2	EB [14, 16]
150 min/week MPA or 75 min/week VPA	2	IPS [18, 20]
30 min MPA 5 d/week, 20 min VPA 3 d/week, or 600 MET-min/week	7	IPS [21], WHO [4, 22, 23], WHS [28–30]
420 min/week MPA or 210 min/week VPA	1	IPEN [26]
5 days/week 30 min MVPA	2	[33] (NI) [36]
150 min/week MPA or 60 min/week VPA	1	[33] (I&SC)
30 min/day MVPA	1	[34]

*EB* Eurobarometer, *IPS* International Prevalence Study, *WHO* World Health Organization, *EPIC* European Prospective Investigation into Cancer and Nutrition, *IPEN* International Physical activity and the Environment Network, *WHS* World Health Survey, *IPAQ* International Physical Activity Questionnaire, *ENERGY* EuropeaN Energy balance Research to prevent excessive weight Gain among Youth, *MET* Metabolic Equivalent, *MPA* Moderate Physical Activity, *VPA* Vigorous Physical Activity, *MVPA* Moderate-to-Vigorous Physical Activity, *PA* Physical Activity, *min* minutes, *d* days, *NI* Northern Ireland, *I* Ireland, *SC* Scotland

## Discussion

In this systematic literature review we aimed to provide an overview of the existing cross-European studies on physical activity levels in adults, to describe the variation in population levels of physical activity in European adults, and to discuss the impact of assessment methods. A total of twenty-five eligible articles were identified, reporting on sixteen different studies. The IPAQ-short questionnaire was used most frequently as assessment method, and the percentage of participants who either were or were not meeting the physical activity recommendations was reported most often as an outcome, with the percentage of participants meeting the physical activity recommendations ranging from 7 % to as high as 96 % across countries and studies.

Some of the (mostly Eastern) countries within the Council of Europe are currently not represented in cross-European surveys and studies on physical activity in adults. Future studies should include these countries in order to gain a complete picture of the population levels of physical activity in all countries across Europe, and to enable comparison and benchmarking.

A variety of questionnaires were used to assess physical activity levels. Although frequently used in physical activity research, subjective self-report measures like questionnaires have well-known limitations such as recall- and social desirability bias [38], limiting their validity. In addition, participants from different countries and/or cultures may interpret questions differently. These limitations do not apply to studies using objective assessment methods like accelerometers. Even though accelerometers have different limitations, such as higher costs and the lack of contextual information, they provide more valid and comparable physical activity data. Two of the identified studies used accelerometers to assess physical activity levels across Europe [26, 27, 37]. One of these studies was conducted in a small sample of young adults in two countries [37], while the other included non-representative samples of adults in five European countries, as part of a larger international study [26, 27]. Therefore, it can be concluded that there is currently a limited amount of objective measurement of physical activity levels in adults across European countries. It should be noted, however, that accelerometer data are available in large scale national representative adult samples from five European countries [39], but these were not included in the current review as they were single-country studies.

Because the different studies used different assessment methods, it is difficult to compare their results. In addition, even within the studies that used the same assessment method, there is substantial variation in the reported outcomes, and even within the studies that report the same outcome, there is variation in the operationalization of that outcome. Moreover, differences in study samples (and non-population representative study samples) might add to this variation. This heterogeneity arguably becomes most apparent in Fig. 2. Even though all of these articles report the percentage of participants meeting the physical activity recommendations (most of them based on questionnaire research), those percentages differ greatly between studies and within countries. This means that population levels of physical activity in European adults are currently unknown.

The methodological variation in the current cross-European studies makes it difficult to compare the population levels of physical activity in terms of absolute numbers. However, since the geographical patterns seem to be reasonably consistent across studies, the identified studies might provide an judicious estimate of the relative order of the countries within Europe, providing the opportunity to identify and target those countries that consistently show the lowest population levels of physical activity. Acknowledging the inconclusive nature of this inference, these countries might include Ireland, Italy, Malta, Portugal, and Spain.

### Strengths and limitations

This systematic literature review is the first - to our knowledge - to provide an overview of all available studies reporting on the population levels of physical activity in adults across Europe. The main strength of this review is the thorough and systematic review process. A review protocol was written before the search was conducted, and adhered to throughout the review process. Combining the search for the four different reviews reduced the chances of missing articles, for example articles that did not explicitly define their target population. The search was performed in six databases, including a database specialized in grey literature, and several additional search strategies were used. In addition, the article selection, data extraction and quality assessment were all conducted by two independent researchers.

Even though the search was performed in several databases and supplemented by additional search strategies, the possibility remains that articles have been missed. Related to this, the fact that we only included articles published in the English language might also have led to missing articles, although cross-European studies are likely to have been published in English.

For these systematic literature reviews, we chose to only include studies that included at least two European countries, because a 2010 WHO report had already identified all national surveillance systems and concluded that their results were not comparable between countries. Hence, national studies were excluded even if objective data was collected, which might have been better comparable. Although, comparisons of such national objective studies might have remained problematic due to differences in data processing across studies. Pooling, harmonizing and comparing available objectively measured national population based physical activity data across Europe might be an opportunity worth exploring in future studies.

Of the twenty-five articles that were eligible for inclusion in this review, several reported on the same study sample. We decided to select one article per study to describe the reported physical activity levels, to avoid presenting results from the same dataset twice. We selected these articles based on the information presented in the article and their similarities with the other articles. Admittedly, these selection criteria are arbitrary. However, because the articles were based on the same data, it is not likely that including different articles would have resulted in different conclusions.

### Results of joint reviews

This review was part of a cluster of four reviews, focusing on the variation in population levels of 1) sedentary time in youth [7], 2) sedentary time in adults [8], 3) physical activity in youth [9], and 4) physical activity in adults (the current review). In adults, more articles reported on physical activity than sedentary time, while this was the other way around for the youth articles. The youth reviews identified a larger number of articles for both behaviours, indicating cross-European studies are more often conducted in youth than in adults. The studies in adults and the sedentary time studies in youth predominantly used questionnaires, while the youth studies on physical activity used accelerometers more frequently. All four reviews displayed substantial variation in the assessment methods used and the reported outcome variables across studies, limiting their comparability.

### Implications

The results of these reviews highlight the need for harmonisation and standardisation of the measurement methods used to assess population levels of physical activity in European countries, as these levels are currently unknown. Ideally, a cross-European surveillance system should be set up, with regular and state-of-the-art measures of physical activity and sedentary behaviour (and their determinants) in youth and adults across Europe. Including objective measures such as accelerometers will provide more valid and comparable estimates of physical activity levels, but might be challenging on such a large scale. Such a surveillance system could ensure the availability and continuity of high-quality data and involve those countries that are currently absent in studies. This could be set-up by harmonising the existing national surveys, integrating these measures in the existing international studies, or setting up a new cross-European monitoring system. The results of these surveillance efforts could be used to inform targeted interventions and public health campaigns, ultimately aiming to increase physical activity levels across Europe.

## Conclusion

A valid overview of adult physical activity levels across Europe is currently lacking. Because of the large variety in the assessment methods used to assess physical activity, the reported outcome variables and the presented physical activity levels per study, absolute physical activity population levels in European adults are currently unknown. When ranking countries with available data, Ireland, Italy, Malta, Portugal, and Spain seemed to report less physical activity, but given the methodological limitations of such comparisons between countries, this observation should be treated with caution. Objective data in adults from cross-European studies is currently limited. These findings highlight the need for standardisation of the measurement methods and data processing used to assess physical activity in Europe, and the added value of a cross-European surveillance system including state-of-the-art physical activity measurements.

## Abbreviations

BMI, body mass index; CS, cross-sectional; DEDIPAC, determinants of diet and physical activity; EB, eurobarometer; Edu, education; EN, England; ENERGY, EuropeaN energy balance Research to prevent excessive weight Gain among Youth; EPIC, European prospective investigation into cancer and nutrition; ESS, European social survey; EUPASS, European physical activity surveillance system; EYHS, European youth heart study; F, Females; GB, Great Britain; H, hours; IPAQ, international physical activity questionnaire; IPEN, international physical activity and the environment network; IPS, international prevalence study; LT, longitudinal; M, Males; MET, metabolic equivalent; Min, minutes; MPA, moderate physical activity; MVPA, moderate-to-vigorous physical activity; n.r, not reported; RoC, Republic of Cyprus; TCC, Turkish cypriot community; VPA, vigorous physical activity; WHO, World Health Organization; WHS, World Health Survey; Yrs, years.

## Additional files

Additional file 1: The PRISMA checklist. (PDF 212 kb) Additional file 2: The complete search string. (PDF 210 kb) Additional file 3: Data extraction file. (XLSX 40 kb) Additional file 4: Quality assessment file. (XLSX 14 kb)
