# Supplementary material for: Variation in population levels of physical activity in European adults according to cross-European studies: a systematic literature review within DEDIPAC
Source: Int J Behav Nutr Phys Act. 2016 Jun 28;13:72. doi: 10.1186/s12966-016-0398-2 (PMC4924233; doi:10.1186/s12966-016-0398-2)
Supplement: Additional file 2: — The complete search string. (PDF 210 kb) [file 12966_2016_398_MOESM2_ESM.pdf]

Additional File 2. The complete search string

#1 ‘Physical activity’ OR ‘Physical activities’ OR ‘Physically active’ OR ‘Physical exercise’

OR

#2 Sedentary OR Sedentari* OR Sitting OR ‘Physical inactivity’ OR ‘Physically inactive’ OR ‘Screen time’ OR Television OR TV OR ‘Video game’ OR ‘Video games’ OR Videogame OR Videogames OR ‘Video-game’ OR ‘Video-games’ OR Gaming OR ‘Computer use’ OR ‘Computer time’

AND

#3 ‘Pan-European’ OR Europe* OR Albania OR Andorra OR Armenia OR Austria OR Azerbaijan OR Belgium OR Bosnia OR Herzegovina OR Bulgaria OR Croatia OR Cyprus OR Czech OR Denmark OR Estonia OR Finland OR France OR Georgia OR Germany OR Greece OR Hungary OR Iceland OR Ireland OR Italy OR Latvia OR Liechtenstein OR Lithuania OR Luxembourg OR Malta OR Moldova OR Moldavia OR Monaco OR Montenegro OR Netherlands OR Norway OR Poland OR Portugal OR Romania OR ‘Russian Federation’ OR ‘San Marino’ OR Serbia OR Slovak* OR Slovenia OR Spain OR Sweden OR Switzerland OR Macedonia OR Turkey OR Ukraine OR ‘United Kingdom’ OR UK OR International

AND

#4 ‘Multi-country’ OR Countries OR Nations OR ‘Member States’ OR International
